# A senescent cell bystander effect: senescence-induced senescence

**DOI:** 10.1111/j.1474-9726.2012.00795.x

**Published:** 2012-04

**Authors:** Glyn Nelson, James Wordsworth, Chunfang Wang, Diana Jurk, Conor Lawless, Carmen Martin-Ruiz, Thomas von Zglinicki

**Affiliations:** 1 Institute for Ageing and Health, Newcastle University, Campus for Ageing and VitalityNewcastle upon Tyne NE4 5PL, UK; 2 Department of Life Science, Walton Hall, Open UniversityMilton Keynes, MK7 6AA, UK

**Keywords:** aging, DNA damage, 53BP1, GFP, fluorescence, cell signalling

## Abstract

Senescent cells produce and secrete various bioactive molecules including interleukins, growth factors, matrix-degrading enzymes and reactive oxygen species (ROS). Thus, it has been proposed that senescent cells can damage their local environment, and a stimulatory effect on tumour cell growth and invasiveness has been documented. However, it was unknown what effect, if any, senescent cells have on their normal, proliferation-competent counterparts. We show here that senescent cells induce a DNA damage response, characteristic for senescence, in neighbouring cells via gap junction-mediated cell–cell contact and processes involving ROS. Continuous exposure to senescent cells induced cell senescence in intact bystander fibroblasts. Hepatocytes bearing senescence markers clustered together in mice livers. Thus, senescent cells can induce a bystander effect, spreading senescence towards their neighbours *in vitro* and, possibly, *in vivo*.

Cellular senescence is a state of irreversible cell cycle arrest, typically driven by a persistent DNA damage response (DDR) (Campisi & d’Adda di Fagagna, 2007). Senescent cells activate downstream signalling pathways that trigger production and release of a host of bioactive molecules including reactive oxygen species (ROS) (Passos *et al.*, 2010) and a wide variety of pro-inflammatory cytokines, chemokines and growth factors (Coppe *et al.*, 2008). Thus, senescent cells stimulated proliferation and invasiveness of premalignant and malignant epithelial cells in co-culture and co-transplantation experiments (Krtolica *et al.*, 2001; Bavik *et al.*, 2006; Liu & Hornsby, 2007). However, the impact of senescent cells upon normal cells with intact DNA damage checkpoints has not been examined.

We hypothesized that pro-oxidant and pro-inflammatory signals from primary senescent founder cells may trigger DNA damage and premature senescence in surrounding primary cells, similar to the classical bystander effect as induced by ionizing radiation (Prise & O’Sullivan, 2009). If so, this senescence-induced bystander effect may contribute to the increasing frequency of senescent cells with age and to the impact senescent cells may have upon their environment.

To test this hypothesis, we examined the effects of co-culturing replicatively senescent fibroblasts (founder cells) with young (recipient/bystander) fibroblasts *in vitro*. We followed the recipient cells by stably integrating a fluorescent fusion protein (AcGFP-53BP1c), which quantitatively reports the number of DNA double-strand breaks (DSBs) within a cell at any given time (Nelson *et al.*, 2009).

Reporter fluorescence in senescent cells differs from their young, replication-competent counterparts in various aspects: (i) Senescent cells display larger numbers of DSB foci (Fig. 1A). In senescent primary human fibroblasts, this amounts to an average of 4–5 DSBs at any one time within the population, compared to a maximum of 1–2 foci in proliferating cells (Fig. 1B). (ii) DNA damage foci in senescent cells are larger (Rodier *et al.*, 2011). Separating 53BP1-AcGFP foci by size, we found that > 90% of senescent MRC5 fibroblasts, but fewer than 50% of young, contained at least one large focus (Fig. S1). (iii) Senescent cells contain long-lived, potentially persistent foci (Passos *et al.*, 2010). Large foci size is associated with long foci lifespan (Fig. 1C). Therefore, foci kinetic data are ideal markers to follow the induction of a senescent phenotype *in vitro*.

**Fig. 1:**
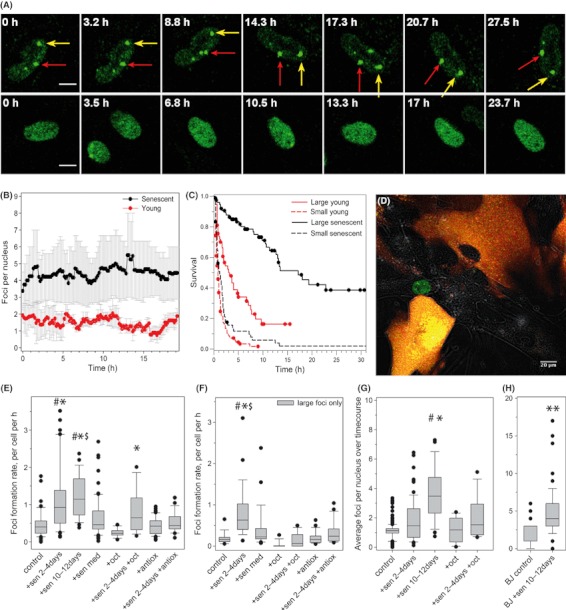
Senescent founder cells induce a senescence-like DDR in bystander cells. (A) Representative images of 53BP1 reporter fluorescence in a senescent (top) or young (bottom) MRC5 cell nucleus with time. Yellow and red arrows indicate two large foci that remain stable over > 27.5 h in the senescent cell. Images are compressed z stacks over 4.5 μm to capture the entire nuclear volume. (B) Mean 53BP1 foci frequencies over time in proliferating (red) and senescent (black) MRC5 cells. Data are mean ± SD of at least 25 nuclei, from three independent experiments. (C) Kaplan–Meier survival curves (censored data) for large (solid lines) and small (dotted lines) 53BP1 foci in proliferating (red) and senescent (black) MRC5 fibroblasts. Foci numbers are 202 (young) and 138 (senescent) from two independent experiments. (D) Representative image of co-cultured senescent founder cells (marked by cytoplasmic RFP staining) and bystander MRC5-AcGFP-53BP1 cells (green nuclear fluorescence). Note nuclear foci in the central bystander cell. (E) 53BP1 foci formation rates in bystander MRC5-AcGFP-53BP1 cells. Co-culture was with no cells without (control) or with either octanol (+oct) or 100 IU SOD and 100 IU catalase (+antiox), with senescent MRC5 cells for 2 days (+sen 2–4 day) or 10 days (+sen 10–12 day) without or with either octanol (+sen 2–4 day +oct) or 100 IU SOD and 100 IU catalase (+sen 2–4 day +antiox) prior to 55 h imaging, or grown in senescent cell conditioned medium for 2 days (+sen med). Box plots indicate median, upper and lower quartiles (boxes), upper and lower centiles (whiskers) and outliers (dots). Significant differences between bystanders and their respective controls (control, +oct or +antiox) are indicated by *, # and $ denote significant differences to antioxidant- or octanol-treated bystanders, respectively (Kruskal–Wallis anova on ranks with Dunn’s *post hoc* test, *P* < 0.05). (F) Formation rate of large foci after 2 days co-culture. Data and statistics are as in (D). (G,H) Average foci frequencies per nucleus in bystander MRC5-AcGFP-53BP1 (G) and BJ-AcGFP-53BP1 (H) fibroblasts. Data and statistics are as in (E).

We next seeded unlabelled or RFP-labelled senescent founder cells in a 1:1 ratio with AcGFP-53BP1-expressing recipient fibroblasts at a PDL of 25 (Fig. 1D). Foci formation rates in young recipient MRC5 cells were significantly increased after short (2 day) exposure to senescent cells and remained elevated during prolonged co-culture (Fig. 1E). This increase in focus induction was exclusively because of more frequent generation of large 53BP1 foci (Fig. 1F); induction rates for small foci were not changed (Fig. S2). Steady-state foci frequencies per nucleus started to increase in the recipient cells after short exposure to senescent cells and reached levels similar to those found in senescent cells after 10 days exposure (Fig. 1G). This was confirmed in an independent foreskin fibroblast strain, BJ (Fig. 1H). These results indicate that senescent human fibroblasts produce a DDR as bystander effect in surrounding proliferating cells.

We performed a series of experiments to clarify how the bystander effect was mediated. Conditioned medium from senescent fibroblasts alone was not sufficient to increase the foci formation rate (Fig. 1E,F) or the steady-state foci frequencies (Fig. S3). Senescent inducer and young recipient cells were also grown as separate layers sharing the same medium in Transwell inserts. Growth in such vessels produced no observable effects in terms of DNA damage foci numbers (data not shown) and had no significant effect on growth rate of the recipient cells over 24 days (Fig. S4). To address the role of the extracellular matrix generated by senescent founder cells, we compared DNA foci frequencies in young recipient cells grown for 7 days on matrix deposited by either young or senescent cells. There was no effect of the matrix on frequencies of either small or large foci (Fig. S5). On the contrary, blocking gap junction-mediated cell–cell contact or scavenging extracellular ROS blocked the increase of foci formation rate (Fig. 1E,F) and steady-state foci levels (Fig. S3) in bystander cells. This is reminiscent of the weak bystander effect induced by low dose and/or low LET irradiation, which is also dependent on direct cell–cell contact via gap junctions (Gaillard *et al.*, 2009) and typically involves signalling through oxygen- and/or nitrogen-centred radicals (Chen *et al.*, 2009).

Senescent cells produce and secrete a variety of candidate signalling molecules, including ROS (Passos *et al.*, 2010), pro-inflammatory interleukins (Kuilman *et al.*, 2008), TGFβ1 (Debacq-Chainiaux *et al.*, 2005) and various IGF-binding proteins (Kim *et al.*, 2007; Muck *et al.*, 2008) that can all maintain or induce senescence in a cell autonomous or nonautonomous fashion. Our data show that any long-lived soluble factors released by senescent cells into the medium or the matrix have little effect on the formation of DNA damage foci. However, such factors could be transmitted between cells via gap junctions.

While our data indicate that ROS are necessary for the induction of DNA damage in the recipient cells, they do not allow the conclusion that ROS released from senescent cells are directly or indirectly causal for the damage in the recipients. Enzymatic antioxidants are essentially confined to the extracellular space including the outer cell membrane and will thus primarily suppress ROS in the medium. However, various ROS species are readily interchangeable, and hydrogen peroxide is easily membrane permeable, so that extracellular antioxidants will effectively reduce intracellular ROS concentrations. Our data are therefore fully compatible with the idea that ROS production in the recipient cells is activated by some unspecified signal(s). However, they strongly suggest ROS as the proximal cause of DNA damage in the bystander cells.

Normal cells with noncompromised DNA damage checkpoint function are expected to react to persistent DNA damage by induction of a senescence or apoptosis programme. To see whether the bystander effect actually induced cell senescence, we measured multiple markers of senescence in the recipient cells. Recipient MRC5 cells proliferated significantly slower (Fig. 2A) and were less positive for the cell cycle marker Ki67 (Fig. 2B), as were recipient BJ cells (Fig. S6). The frequency of bystander cells positive for senescence-associated β-galactosidase (Sen-β-Gal) activity was increased after co-culture for 15 or more days (Fig. 2C). It was measured after removal of senescent inducer cells from the culture, showing that the bystander effect induced permanent senescence. After extended co-culture with senescent cells, MRC5 and BJ bystander cells showed stronger p38MAPK activation (Fig. 2D) and more frequent nuclear PML:γH2AX co-localization (Figs 2E and S7), two additional markers of senescence. Together, these data show that senescent cells induce permanent cell senescence as a bystander effect in their environment.

**Fig. 2:**
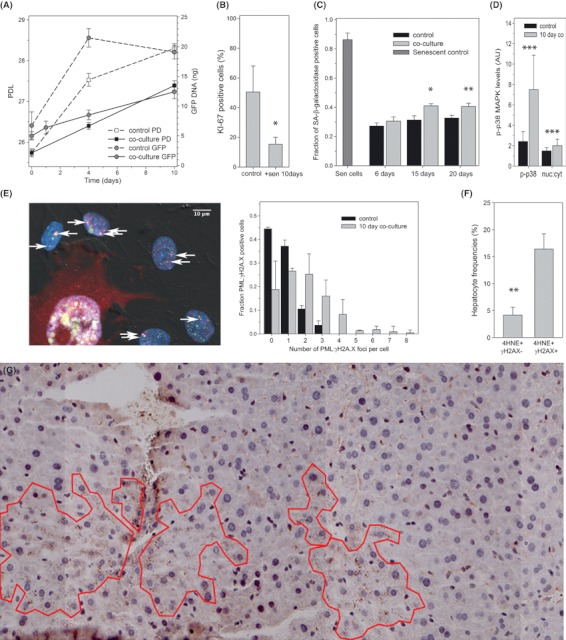
Senescent cells induce senescence in surrounding cells. (A) Impact of co-culture with senescent MRC5 cells on growth of MRC5-AcGFP-53BP1 cells measured by either cell counts (square symbols) or GFP-qPCR (circles). Cells were not replated during the experiment, leading to some density-mediated growth inhibition in control cells towards the end of the experiment. However, growth of bystander cells in co-culture was significantly slower (*P* < 0.01 at 4 and 10 days). (B) Frequencies of Ki67-positive MRC5-AcGFP-53BP1 cells after 10 day culture on their own (control) or 1:1 co-culture with senescent fibroblasts. Data are mean ± SEM, *n* = 4, *P* = 0.047. (C) Frequencies of Sen-β-Gal-positive MRC5-AcGFP-53BP1 bystander cells after the indicated time of co-culture with or without (control) senescent MRC5 cells. Senescent inducer cells were removed by blasticidin treatment for 6 days. Increases after 15 and 20 days co-culture are significant (anova with Tukey HSD *P* = 0.048 and 0.008, respectively). (D) Phospho-p38MAPK (P-p38) levels and nuclear:cytosolic ratios (measured by immunofluorescence) in MRC5-AcGFP-53BP1 after 10 days co-culture. Data are mean ± SD, *P* < 0.001 (*T*-test). (E) Co-localization (yellow) of γH2AX foci (green) with PML bodies (red) in bystander BJ-AcGFP-53BP1 cells after 10 day co-culture with senescent cells (marked by red RFP cytoplasmic fluorescence). Left: representative image; right frequency distributions of co-localization-positive cells. Means and distributions are significantly different (*P* < 0.0000, Mann–Whitney *U*-test). (F) Percentage of hepatocytes in adult mice liver showing cytoplasmic 4HNE staining together with (4HNE+ γH2AX+) or without (4HNE+ γH2AX−) nuclear DDR. Data are mean ± SE, *n* = 4 animals. (G) Representative tiled image of mouse liver (9 months old) immunostained for 4-HNE. Areas of clustered 4-HNE-positive cells are outlined. DDR, DNA damage response.

If the bystander effect were important for the generation of cell senescence *in vivo*, senescent cells would be expected to cluster in tissues. We chose mouse liver for cluster analysis as a relatively homogeneous tissue with a significant fraction of senescent hepatocytes (Krizhanovsky *et al.*, 2008; Wang *et al.*, 2009). We used 4-HNE as a marker for senescent cells because it is closely associated with other markers of senescence in mouse liver including γH2A.X (Fig. 2F) and Sen-β-Gal (Wang *et al.*, 2009, 2010) and allows clear cell boundary definition in the low magnification tiled images necessary for cluster analysis. Marker-positive hepatocytes formed closely associated clusters with essentially no negative cells between them (Fig. 2G). This clustering is significantly higher than expected by random chance, given the observed frequencies of marker-positive hepatocytes in mouse livers (Fig. S8). Such clustering could in principle be driven by focal oxidative damage. However, it should be noted that we did not see evidence for leucocyte invasion associated with clusters of 4-HNE-positive cells. While comprehensive proof of senescence-induced senescence in tissues awaits the analysis of a senescence reporter system *in vivo*, our data already indicate that senescent cells induce a bystander effect that spreads DNA damage and, ultimately, induces cell senescence in primary, checkpoint-competent cells *in vitro* and, possibly, *in vivo*. This could explain how senescent cells might drive the aging process *in vivo* as proposed (Tchkonia *et al.*, 2010; Baker *et al.*, 2011).

## Supporting Information

**Data S1.** Experimental procedures.

**Figure S1.** Frequencies of cells containing small and/or large AcGFP-53BP1 foci in proliferating and senescent MRC5 cultures.

**Figure S2.** AcGFP-53BP1 small foci formation rates do not differ with treatments.

**Figure S3.** Average foci frequencies per nucleus in bystander MRC5-AcGFP-53BP1 cells.

**Figure S4.** Growth curves of proliferating MRC5-AcGFP-53BP1 cells co-cultured with senescent MRC5 cells or no cells in Transwell dishes.

**Figure S5.** Extracellular matrix does not change DNA damage foci frequencies.

**Figure S6.** Frequencies of Ki67-positiveBJ-AcGFP-53BP1 cells after 10 day culture on their own(control) or 1:1 co-culture with senescent fibroblasts.

**Figure S7.** Frequency distributions ofγH2AX foci colocalising with PML bodies per nucleus inbystander MRC5-AcGFP-53BP1 cells after 20 day co-culture withsenescent cells and in controls.

**Figure S8.** Potentially senescent hepatocytes cluster in ageing mice livers.

As a service to our authors and readers, this journal provides supporting information supplied by the authors. Such materials are peer-reviewed and may be re-organized for online delivery, but are not copy-edited or typeset. Technical support issues arising from supporting information (other than missing files) should be addressed to the authors.
