# Supplementary figures and images for: A senescent cell bystander effect: senescence-induced senescence
Source: Aging Cell. 2012 Apr;11(2):345–9. doi: 10.1111/j.1474-9726.2012.00795.x (PMC3488292; doi:10.1111/j.1474-9726.2012.00795.x)

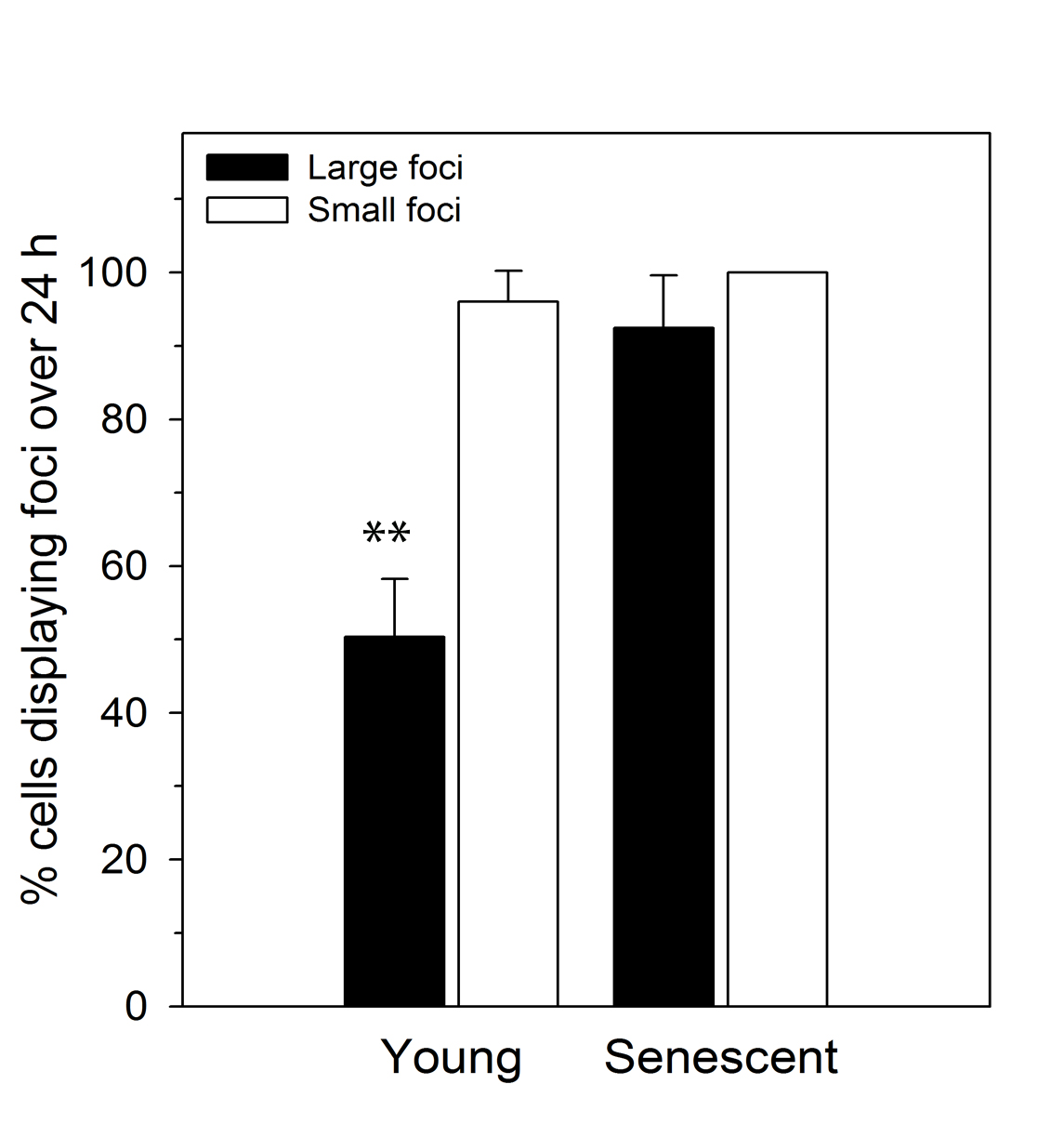

Supplement: Supplementary file 1 [file acel0011-0345-SD1.jpg]

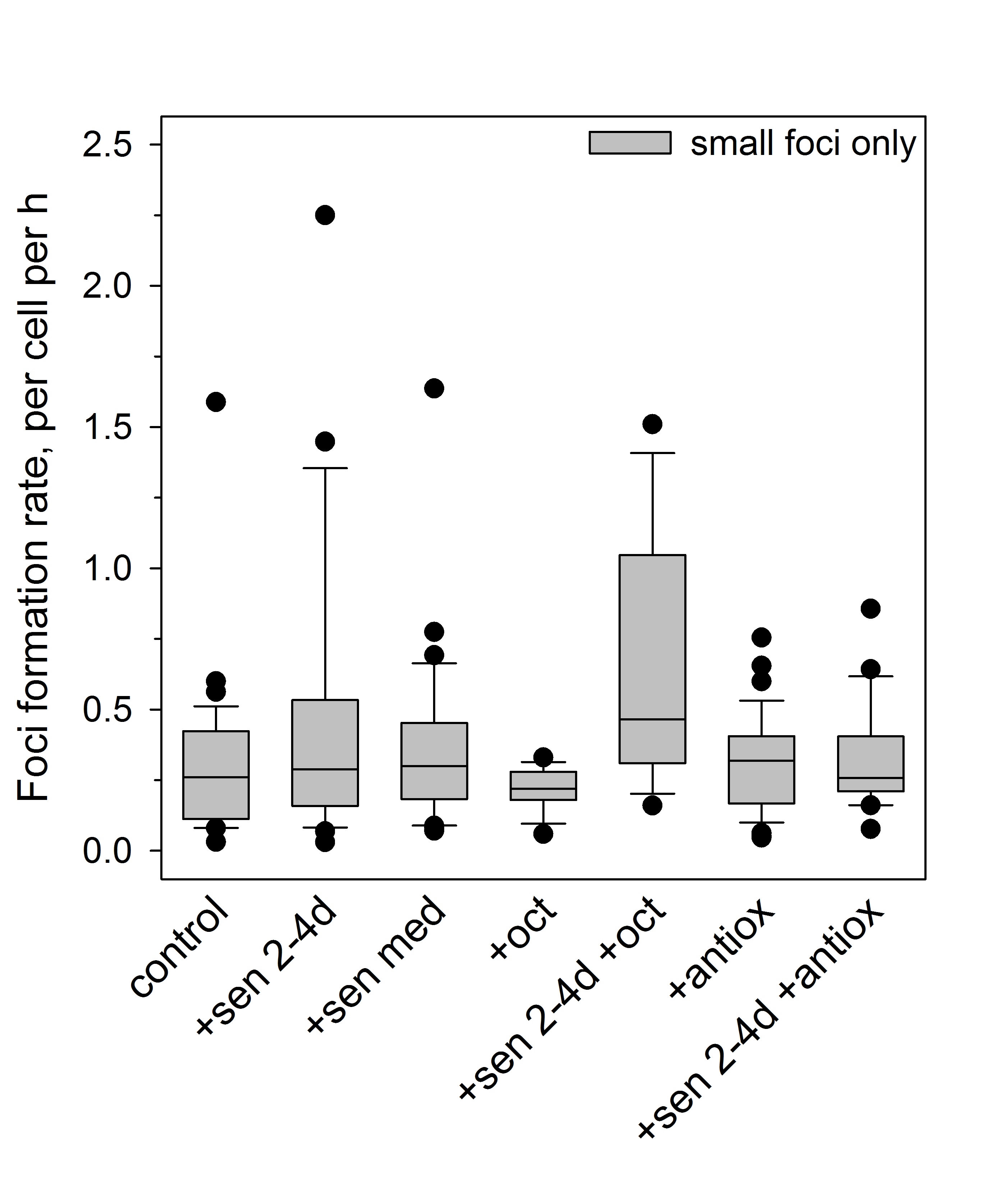

Supplement: Supplementary file 2 [file acel0011-0345-SD2.jpg]

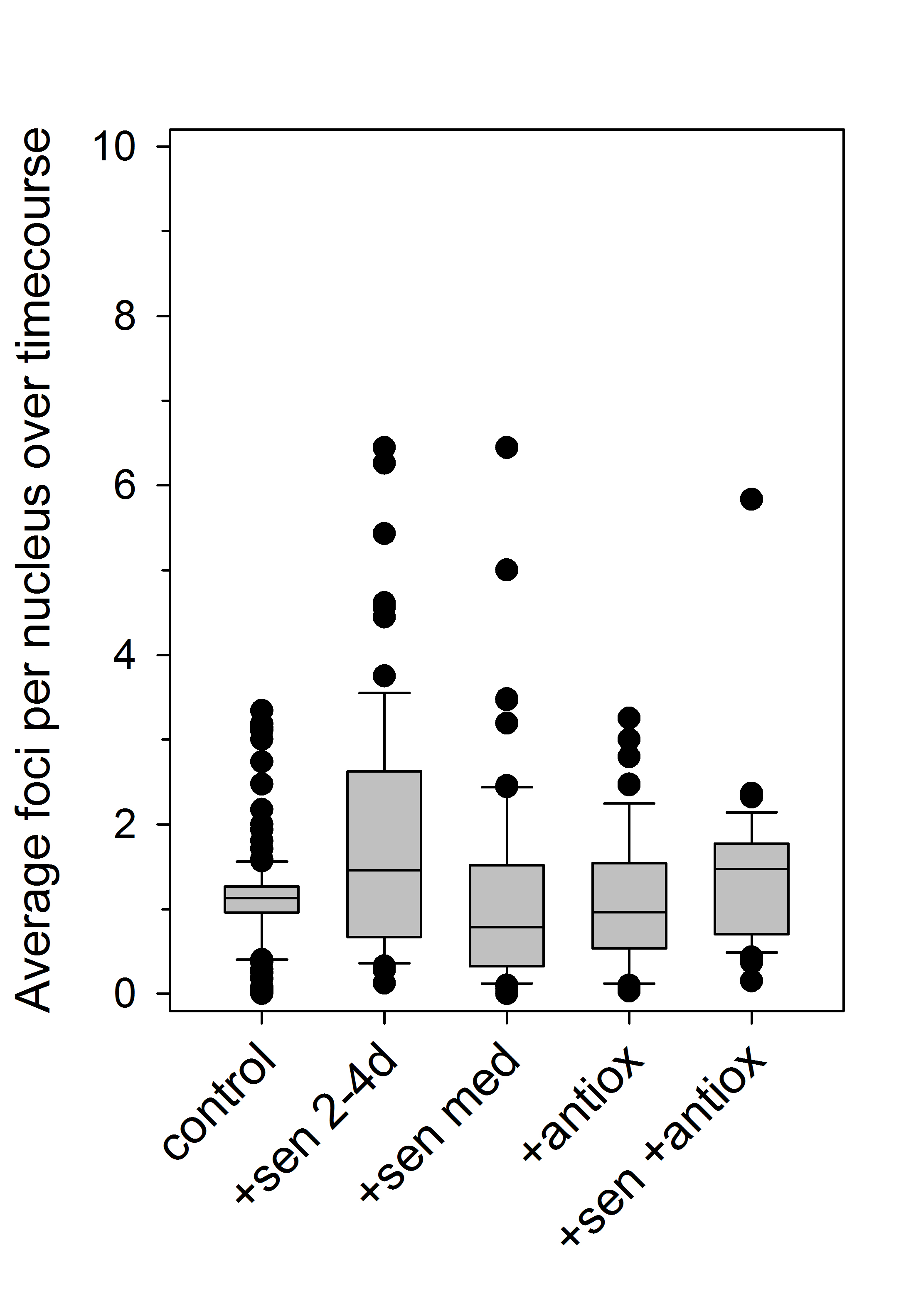

Supplement: Supplementary file 3 [file acel0011-0345-SD3.jpg]

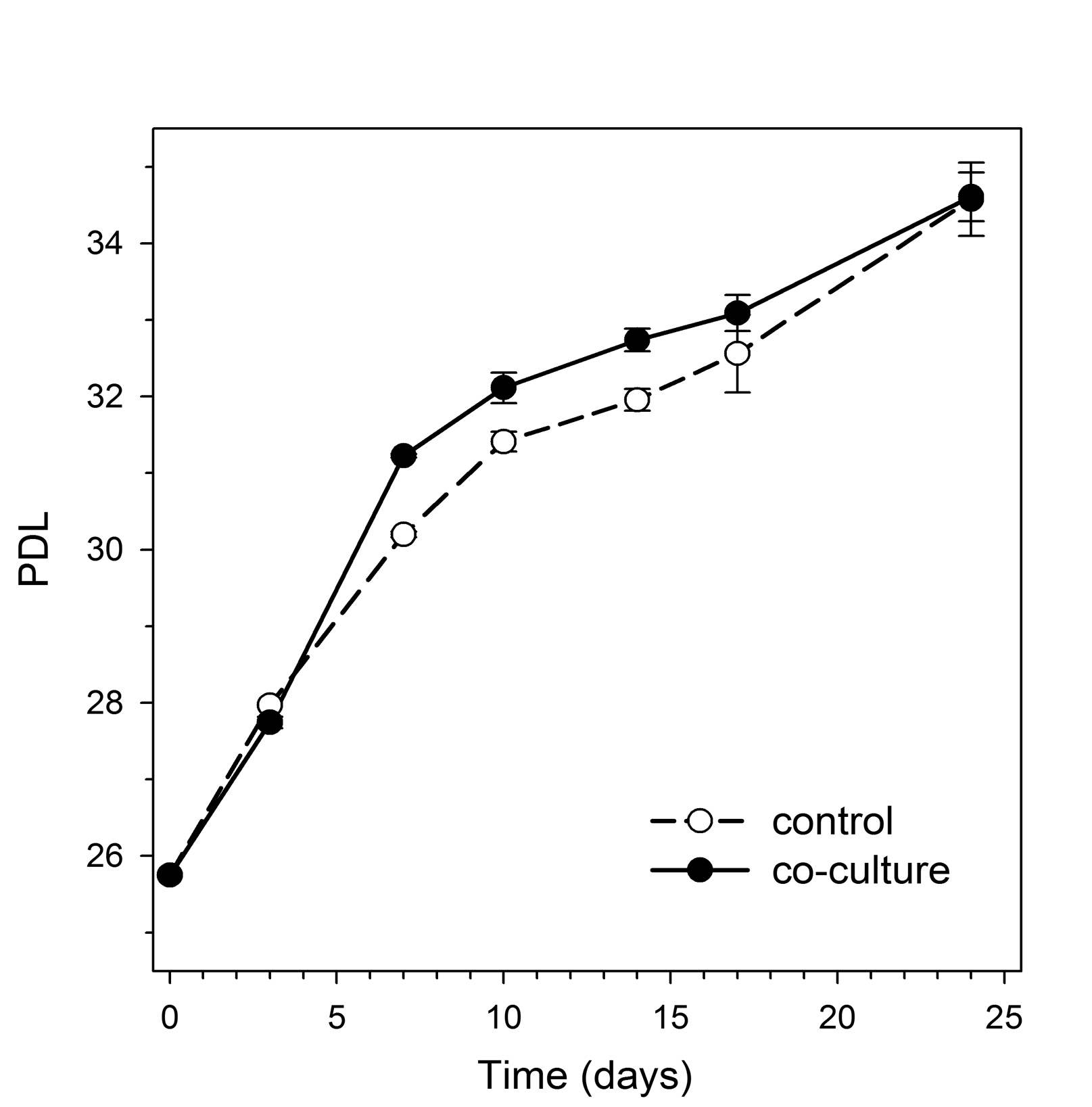

Supplement: Supplementary file 4 [file acel0011-0345-SD4.jpg]

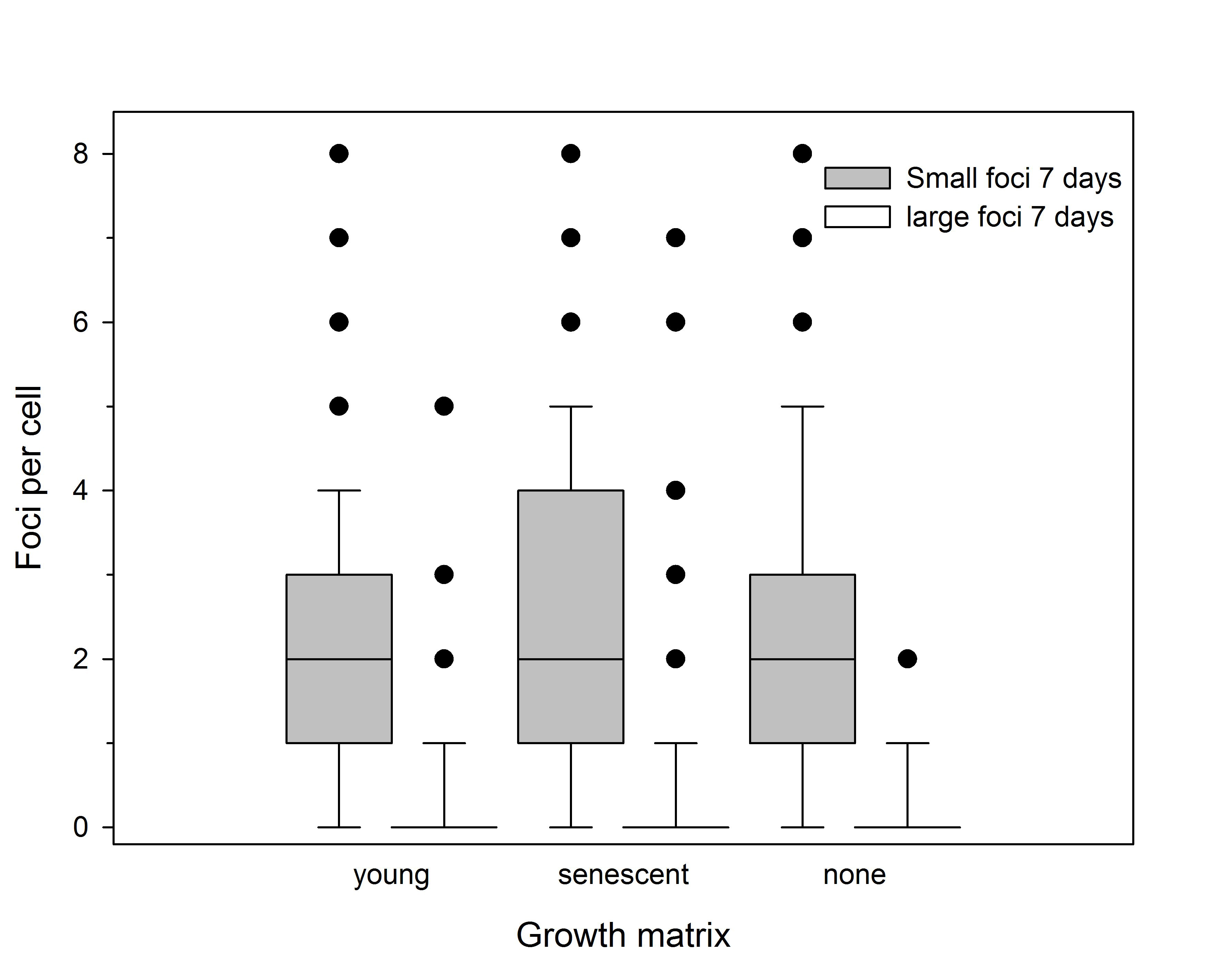

Supplement: Supplementary file 5 [file acel0011-0345-SD5.jpg]

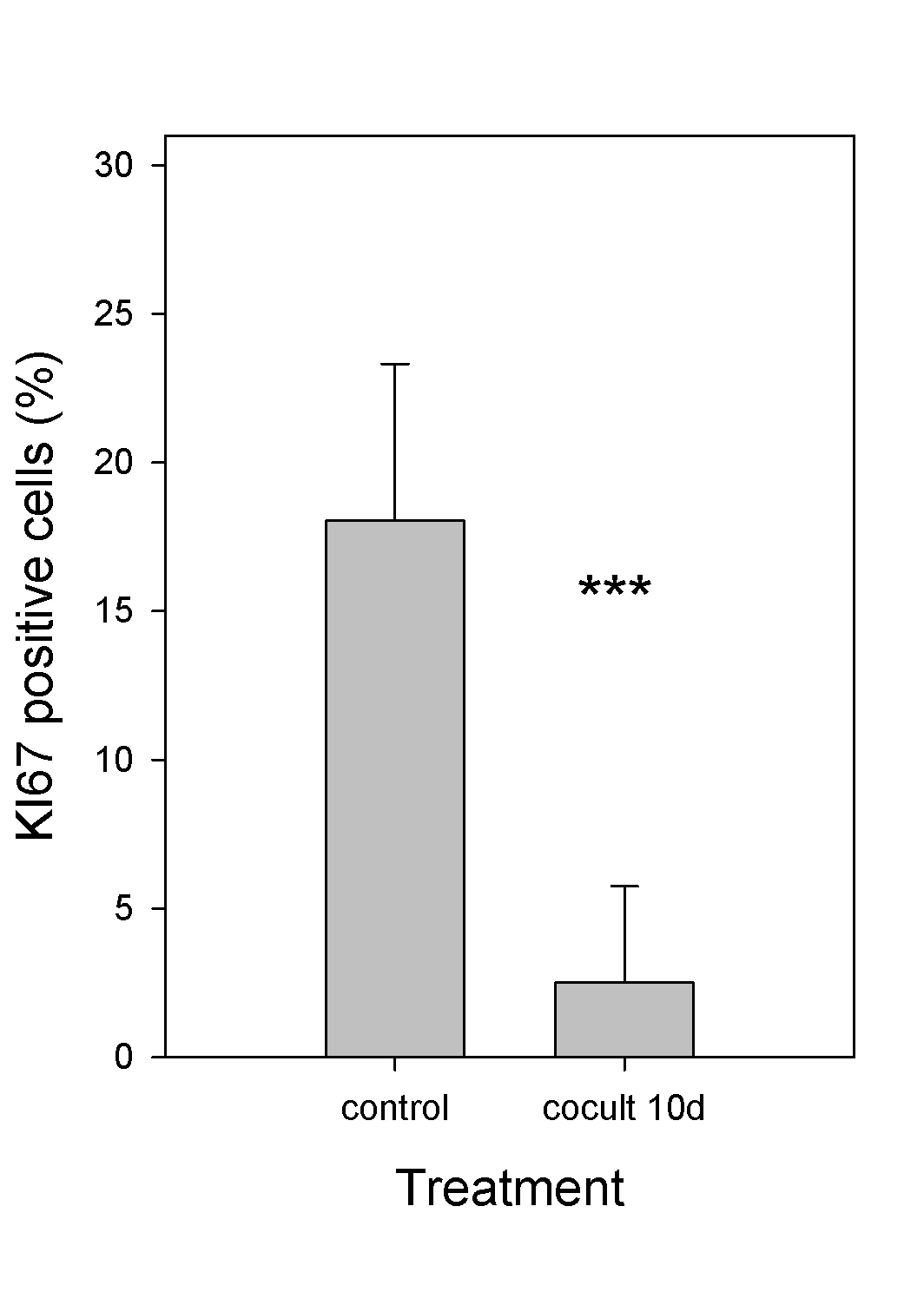

Supplement: Supplementary file 6 [file acel0011-0345-SD6.jpg]

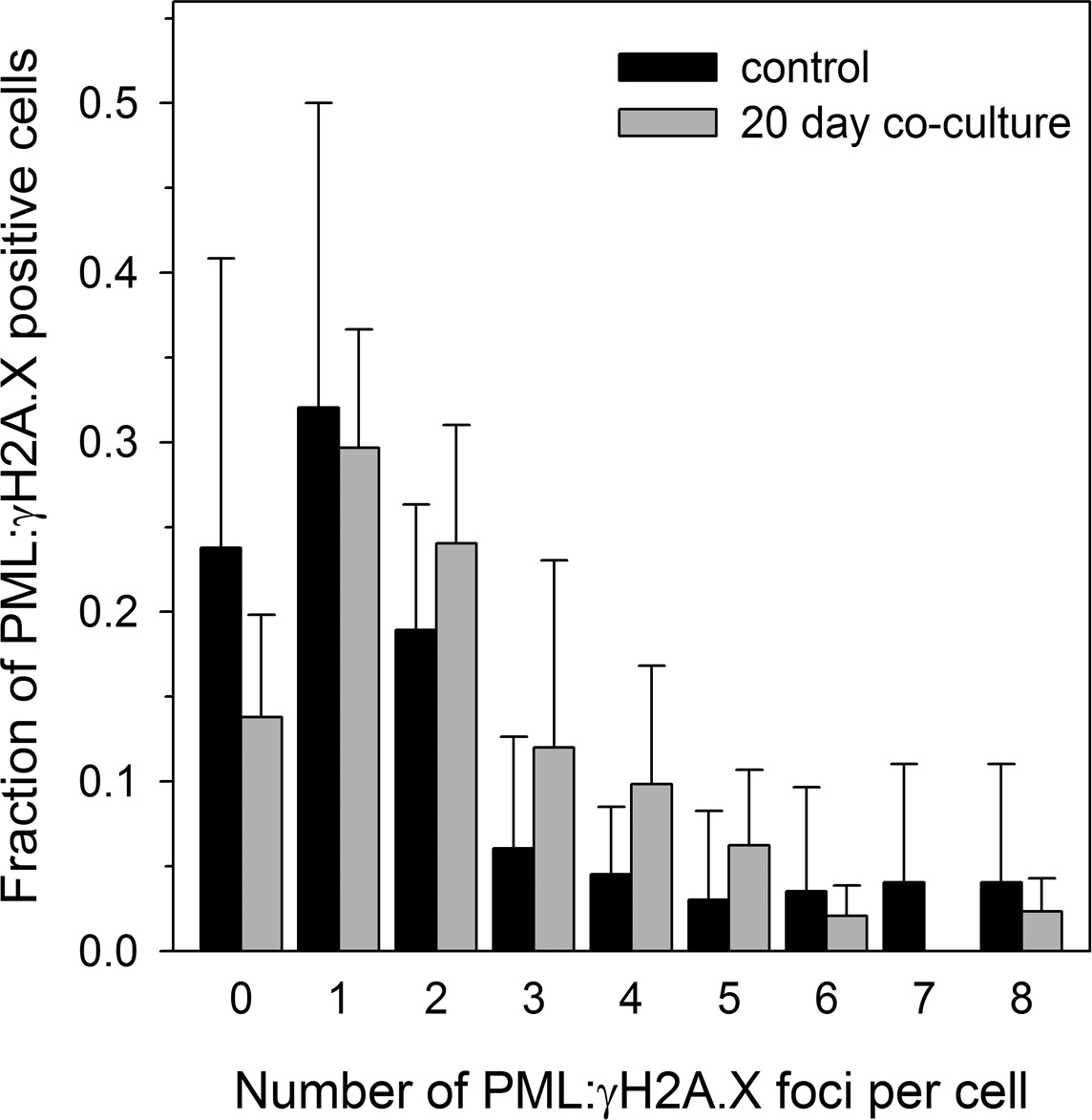

Supplement: Supplementary file 7 [file acel0011-0345-SD7.jpg]

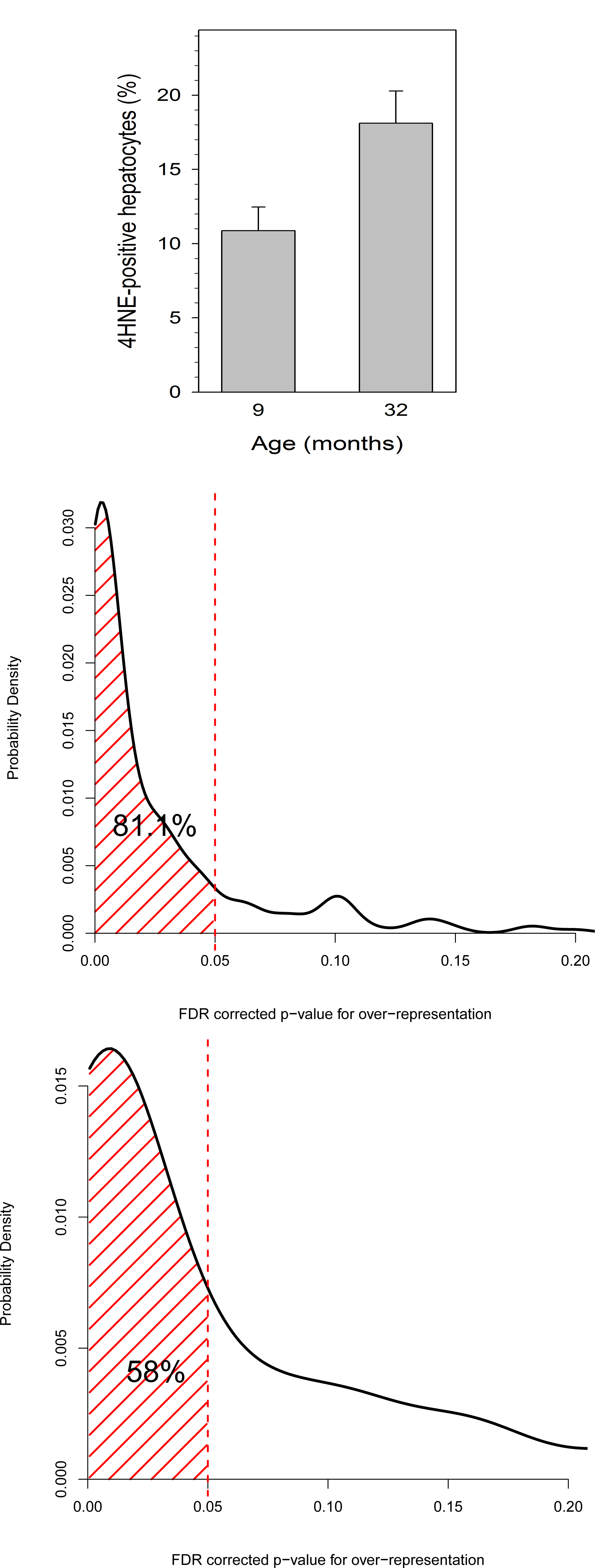

Supplement: Supplementary file 8 [file acel0011-0345-SD8.jpg]
