# Supplementary material for: A senescent cell bystander effect: senescence-induced senescence
Source: Aging Cell. 2012 Apr;11(2):345–9. doi: 10.1111/j.1474-9726.2012.00795.x (PMC3488292; doi:10.1111/j.1474-9726.2012.00795.x)
Supplement: Supplementary file 9 [file acel0011-0345-SD9.doc]

Supplementary Material

## Experimental Procedures

All chemicals were purchased from Sigma Aldrich (Dorset, UK) unless otherwise stated.

**Cells:** Cells were cultured in Dulbecco’s modified Eagle’s medium (DMEM) supplemented with 10% foetal bovine serum (BioSera, Ringmer, UK), 100 units/ml penicillin, 100 μgml^-1^ streptomycin and 2 mM glutamine (complete medium). Cells were grown in a Binder Incubator at 37ºC, 95% air, 5% CO_2_. HEK293FT lentivirus packaging cells were from Invitrogen (Paisley, UK). MRC5 human embryonic lung fibroblasts and BJ human foreskin fibroblasts were from ECACC. Cells were considered replicatively senescent once their growth rate was below 0.1 population doublings (PD) per month. For indirect co-culture, cells were plated in 0.4 µm pore Transwell dishes (Costar, Corning, Amsterdam, Netherlands), with senescent cells plated at confluency onto the membrane and young MRC5 in the dish below at approximately 25% confluency. Direct co-culturing experiments were performed by plating cells subconfluently at a 1:1 ratio. Cell growth rates in these experiments were determined with subtraction of a static senescent population, whose concentration at each passage was re-adjusted to a 1:1 ratio. Confirmation of the PD rate was performed using PCR of gDNA for the integrated GFP fusion protein with the following primers: AcGFP 5'; ATGACGGCAACTACAAGTCG and AcGFP 3'; GTTGCCATCCTCCTTGAAAT. To measure various markers of cell senescence in bystander cells, co-culture was performed for at least 10 days. If co-cultures were terminated after 10 days, cells were not re-plated, so that bystander cells became density-arrested over the course of the experiment. In longer-lasting co-culture experiments, cells were regularly passaged before reaching confluency with re-adjustment of the 1:1 cell ratio at every passage. To positively identify replicatively senescent cells, a red fluorescent protein expressing MRC5 population was created by transduction with RFP (Bsd) lentiviral particles (AMS Biotechnology, Oxford, UK) following the manufacturer’s instructions at a target MOI of 5. Cells were subsequently maintained in selection medium containing 4 μgml^-1^ blasticidin. Cultures were verified to be 100% positive for RFP expression by epifluorescent microscopy before growing to replicative senescence. Gap junction inhibition was performed by treatment of cultures with 1 μM octanol with or without senescent founder cells. Antioxidant treatment was performed with the addition of 100 IU SOD and 100 IU catalase to the medium. Octanol and antixodidant containing medium was refreshed every 2 days.

**Plasmids:** The 3.9 kb AcGFP-53BP1c fusion construct was excised from pG-AcGFP-53BP1c (Nelson *et al.*, 2009) using *Nhe*I and *Sma*I, blunted, and inserted into the *Ehe*I and *Eco*RV sites of pENTR2B (Invitrogen). The resulting plasmid, pE2B-AcGFP-53BP1c was then recombined in a Clonase reaction with pLenti6/UbC/V5-DEST (Invitrogen) to create pLenti-AcGFP-53BP1c. Virions carrying copies of pLenti-AcGFP-53BP1 were created in HEK293FT cells using transfection alongside a plasmid packaging kit following the manufacturer's protocol (Invitrogen). Lentiviral transduction of young MRC5 was carried out using pLenti-AcGFP-53BP1c virions following the manufacturer's protocol with a target MOI of 2. Cells were subsequently maintained in selection medium containing 4 μgml^-1^ blasticidin.

**Microscopy:** For live cell microscopy, cells were plated in glass coverslip bottomed dishes (Iwaki, NELS, Newton Aycliffe, UK) at least one day before utilization, and microscopy was performed as described previously, imaging cells every 10 minutes as a z stack over 4.5 μm (Nelson *et al.*, 2009). AcGFP-53BP1 foci were analyzed as described previously (Nelson *et al.*, 2009). Sen-β-gal staining was performed as described previously (Dimri *et al.*, 1995), after senescent founder cells were removed from the cultures by blasticidin treatment for 6 days and with the addition of DAPI as a counterstain. Immunofluorescence was performed using the following antibodies: PML; Abcam ab53773 (1:250 dilution), γH2AX Upstate, clone JBW103 (1:1250 dilution), total p38; Cell Signalling #9212 (1:200 dilution), phospho-p38; Cell Signalling #9216 (1:400 dilution) using Alexa fluor conjugated secondary antibodies (Invitrogen) at 1:2000 dilution and DAPI as counterstain. Incubation with all primary antibodies was overnight at 4^o^C.

**Quantitative analysis of cluster probability *in vivo*:** 5 μm liver sections from 9 and 32 months old mice (3 animals per age group) were immuno-stained for 4-HNE (clone HNHEJ-2, #MHN-020p, Japan Institute for the Control of Aging, 1:500 dilution) as described (Wang *et al.*, 2010; Wang *et al.*, 2009). 10-20 images per section were taken using a 20x, 0.5NA objective and tiled together. The total number of cells (*N*) and the number of 4-HNE- positive cells (*M*) per tiled image were counted as well as the total number of neighbours (*n_i_*) and the number of 4HNE-positive neighbours (*m_i_*) for each of the positive cells (*i* = *1...M*). To test whether a positive cell was more likely to have positive neighbours than negative neighbours given the prevalence of positive cells in the image (that is, whether positive cells were clustered to a larger degree than expected by random chance), a hyper-geometric test for over-representation of positive cells in the neighbourhood of each positive cell was carried out. A p-value (*p_i_*) for over-representation was calculated for each neighbourhood. The probabilities *p_i,..,M_* were corrected for multiple comparisons by false discovery rate (FDR) control to give q-values (*q_i,...,M_*). The fraction (*F*) of positive cells having significantly more positive neighbours than expected by chance given the background proportion of positive cells observed was estimated as:


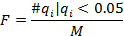


**Other Statistical tests:** All analyses were performed in R (<http://cran.r-project.org/>) unless stated otherwise. Data were tested for normality using Shapiro-Wilk normality test. All non-normally distributed data were analysed via either Kruskal-Wallis for paired tests or Steel's non parametric multiple comparison test. Normally distributed paired data were analysed by Student's T test using OpenOffice (http://www.openoffice.org). Kaplan Meier survival curves were compared using Cox Breslow regression. Significance is denoted on all graphs with * p < 0.05, ** p < 0.01, *** p < 0.001, unless indicated otherwise.

**Supplementary Figure Legends**

**Figure S1. Frequencies of cells containing small and/or large AcGFP-53BP1 foci in proliferating and senescent MRC5 cultures.** Upper size limit was 0.45 μm^2^ for small foci. Data are mean ± SD from 3 experiments, p < 0.001.

**Figure S2. AcGFP-53BP1 small foci formation rates do not differ with treatments.** MRC5-AcGFP-53BP1 cells were treated as described in Experimental Procedures, and rates of foci formation for GFP foci smaller than 0.45 μm^2^ were determined per cell over a 55 h timecourse. Symbols and statistics are as described in Fig. 1E. No significant differences were observed between any of the treatments.

**Figure S3. Average foci frequencies per nucleus in bystander MRC5-AcGFP-53BP1 cells.** Reporter cells were grown on their own without (control) or with 100 IU SOD and 100 IU catalase (+antiox), in senescent cell conditioned medium for 2 days (+sen med) or co-cultured with senescent MRC5 cells for 2 days in the absence (+sen 2-4d) or the presence of the same concentrations of SOD and catalase (+sen +antiox). Box plots indicate median, upper and lower quartiles (boxes), upper and lower centiles (whiskers) and outliers (dots). There were no significant differences between treatments.

**Figure S4. Growth curves of proliferating MRC5-AcGFP-53BP1 cells** co-cultured with senescent MRC5 cells or no cells in Transwell dishes. Data are mean ± SEM, n = 3.

**Figure S5. Extracellular matrix does not change DNA damage foci frequencies.** Coverslips were covered with extracellular matrix by growing confluent young or senescent cells for 1 week followed by hydrolysis in water. Fresh MRC5 cells were then grown for 7 days on plain coverslips or matrices deposited by either young or replicatively senescent cells prior to fixing and staining for DNA damage foci using γ-H2A.X immunofluorescence. Frequencies of large and small foci were analyzed separately. No significant differences between the different growth conditions were observed for either small or large foci. Data are represented as individual cell values from three independent replicates.

**Figure S6. Frequencies of Ki67-positive BJ-AcGFP-53BP1 cells** after 10 d culture on their own (control) or 1:1 co-culture with senescent fibroblasts. Data are mean ± SEM, n = 4, p = 0.006.

**Figure S7. Frequency distributions of γH2AX foci colocalising with PML bodies per nucleus in bystander MRC5-AcGFP-53BP1 cells** after 20 d co-culture with senescent cells and in controls. Means and distributions are significantly different (p = 0.016, Mann-Whitney U test).

**Figure S8. Potentially senescent hepatocytes cluster in ageing mice livers.** A) Frequencies of 4-HNE-positive hepatocytes at the indicated ages. Data are mean ± SEM, n=3, p=0.03. B) and C) Probability density plots for the significance of over-representation of 4-HNE-positive hepatocytes in the neighbourhood of a positive cell in livers from 9 (B) and 32 (C) month old mice. The shaded area indicates the percentage *F* of all cells that have significantly (p = 0.05) more positive neighbours than expected by chance, given the frequency of positive cells in the samples.
